# Research on the mechanism of action of the Li Qi Dao Tan Decoction in the treatment of schizophrenia based on network pharmacology and molecular docking

**DOI:** 10.1097/MD.0000000000043462

**Published:** 2025-07-25

**Authors:** Shuo Wang, Fei Guo, Chen Zhao, Gezi Shi, Jing Liu, Shichang Yang

**Affiliations:** a The Second Affiliated Hospital of Xinxiang Medical University, Xinxiang, China; b Xinxiang Key Laboratory of Traditional Chinese and Western Medicine in Diagnosis and Treatment for Mental and Neurological Diseases, Xinxiang, China; c Clinical Medical Center of Traditional Chinese and Western Medicine for Psychiatric Diseases of Xinxiang Medical University, Xinxiang, China; d Henan Collaborative Innovation Center of Prevention and Treatment of Mental Disorders, Xinxiang, China.

**Keywords:** liqidaotan decoction, molecular docking, network pharmacology, schizophrenia

## Abstract

This study explores the potential targets and signaling pathways of the Li Qi Dao Tan Decoction (LQDTD) in the treatment of schizophrenia using network pharmacology and molecular docking technology. The traditional Chinese medicine systems pharmacology database and analysis platform database was searched to obtain the active ingredients and targets of the LQDTD. Databases such as GeneCards, DrugBank, and OMIM were searched for schizophrenia-related disease targets. By drawing a Venn diagram, the intersection targets of LQDTD and schizophrenia were obtained. The STRING database was used to construct the protein–protein interaction. The online tool of the DAVID database was used to analyze the gene ontology and Kyoto Encyclopedia of Genes and Genomes pathways of the intersection targets of LQDTD and schizophrenia. The Cytoscape software was employed to construct the network of traditional Chinese medicine ingredients, targets, and pathways. AutoDock, Chem3D, and PyMOL software facilitated molecular docking and the calculation of binding energy for the identified key active ingredients and targets, from which the 7 pairs with the lowest binding energy were selected. PyMOL was utilized for analysis and visualization. We identified 5 active ingredients of LQDTD: luteolin, nobiletin, baicalin, quercetin and licochalcone A. Key proteins, including signal transducer and activator of transcription 3, TP53, and EGFR, were identified through the protein–protein interaction map. Kyoto Encyclopedia of Genes and Genomes analysis indicated that the pathways involved primarily included lipid and atherosclerosis pathways, hepatitis B virus-related pathways, AGE-RAGE signaling pathways, Chemical carcinogenesis receptor activation, and prostate cancer signaling pathways. The treatment of schizophrenia may influence key proteins such as signal transducer and activator of transcription 3, TP53, and EGFR, intervening in lipid and atherosclerotic pathways, hepatitis B virus-related pathways, AGE-RAGE signaling pathways, chemical carcinogenesis receptor activation, and prostate cancer signaling pathways. However, the exact mechanisms of action require further experimental verification.

## 1. Introduction

Schizophrenia is a complex of mental disorders marked by disruptions in sensory perception, thought processes, emotions, behavior, and other facets of mental functioning, often resulting in uncoordinated cognitive activities. Individuals with schizophrenia frequently exhibit a lack of self-awareness regarding their condition. The onset of the disorder typically occurs in young adulthood.^[1]^ In TCM, it is classified under the category of “madness.”^[2]^ Before the Jin and Tang dynasties, the condition was referred to as “epilepsy.” Following the Tang Dynasty, descriptions of “madness” emerged. The onset of this condition is understood through 2 perspectives: deficiency syndrome and excess syndrome. The excess syndrome is primarily attributed to pathological factors such as phlegm, fire, and stasis. Which can disrupt the spirit and lead to illness. Chen Xuan et al^[3]^ conducted cluster analysis and principal component analysis, demonstrating that the symptoms of madness were primarily associated with heart and spleen deficiency syndrome, phlegm qi stagnation pattern, liver qi deficiency, and spleen deficiency syndrome, as well as spleen and kidney yang deficiency syndrome.

In the youth stage, the most common condition is the excessive fire injuring yin. In middle-aged and elderly individuals, liver depression with phlegm accumulation is predominant. In later stages, there are often complex conditions characterized by a mix of deficiency and excess syndromes. The earliest record of the phlegm soup appears in the work “Applicable Prescription for Transmission” by the Song Dynasty physician Wu Yanyu.^[4]^ The complete formula consists of Pinelliae Tuber, Arisaematis Rhizoma, Poria, Licorice Root, Fructus Aurantii Immaturus, Red Tangerine Peel, and fresh ginger. Yan Yonghe, who is another physician in the same era, added radix glycyrrhizae preparata to this formulation. Modern clinical studies often utilize the “Phlegm-draining Decoction” recorded in the “Jisheng Fang,” which is the Erchen Decoction modified by removing Dark plum and adding Arisaema heterophyllum Blume and Immature Orange Fruit. This formulation is commonly used to treat conditions caused by qi stagnation and phlegm retention. It is composed of herbal ingredients that effectively soothe the liver, regulate qi, clear turbidity, and strengthen the spleen while transforming phlegm.

Li Qi Dao Tan Decoction (LQDTD) is a classical formula in Traditional Chinese Medicine (TCM), originating from the ancient Song Dynasty, which dates back over 770 years. Based on long-term clinical application, it has shown no significant safety concerns. Our hospital’s TCM pharmacy has dispensed LQDTD for clinical use with inpatients diagnosed with schizophrenia. The Ethics Committee has approved this trial, and it is currently in progress. Furthermore, during the ongoing trial, we regularly monitor patients’ clinical variables and side effects to ensure the medication’s safety.

With the rise of network pharmacology, its holistic and systematic characteristics align with the integrative perspective of TCM and the principles of syndrome differentiation. This study employs network pharmacology methods,^[5–8]^ through data mining and analysis, this study identifies the active components of the LQDTD, as well as potential targets and signaling pathways for the treatment of schizophrenia. The aim is to correlate the active components with protein targets, providing a theoretical basis for the treatment of schizophrenia.

## 2. Materials and methods

**Figure 1.**
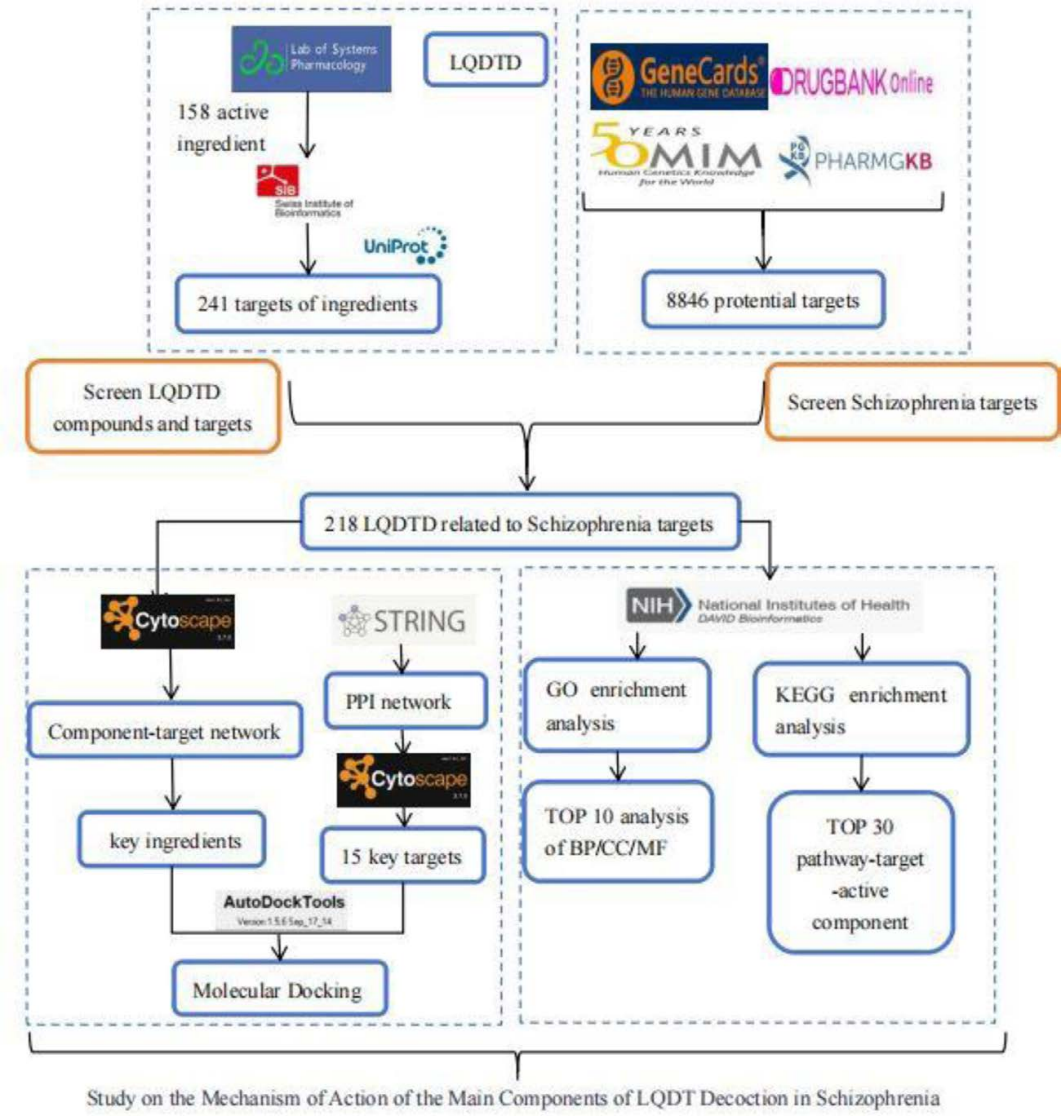
Study on the mechanism of action of the main components of LQDT decoction in schizophrenia. LQDTD = Li Qi Dao Tan Decoction.

The flowchart of this research process is displayed in Figure 1.

### 2.1. Applied database

Traditional Chinese medicine systems pharmacology database and analysis platform (TCMSP; TCM Systems Pharmacology; https://tcmsp-e.com/tcmsp.php); PubChem (https://tcmsp-e.com/tcmsp.php); BATMAN-TCM; Swiss Target Prediction - SIB Swiss Institute of Bioinformatics (expasy.org, https://www.expasy.org/resources/swisstargetprediction); Uniport (https://www.uniprot.org/); OMIM (https://mirror.omim.org/); DrugBank; GeneCards (https://www.genecards.org/); TTD-DatabaseCommons (https://ngdc.cncb.ac.cn/databasecommons/database/id/4948); String (https://cn.string-db.org/); Cytoscape (version 3.9.1); PDB (https://www.rcsb.org/); PyMOL were used.

### 2.2. Active ingredients in LQDTD

Using the TCMSP, we retrieved the ADME parameters for the chemical components related to the 6 TCMs in the LQDTD: Pinelliae Tuber, Arisaematis Rhizoma, Poria, Licorice Root, Fructus Aurantii Immaturus, and Red Tangerine Peel,^[9]^ using oral bioavailability ≥30% and drug-likeness ≥0.18 as screening criteria. The preliminary screening revealed that 158 compounds in the LQDTD met the specified criteria.

### 2.3. Objectives associated with active ingredients

Six TCM targets were identified using the TCMSP database. The UniProt database^[10]^ was utilized to convert the target proteins into their corresponding target genes.

### 2.4. Screening of schizophrenia targets

Using “schizophrenia” as a keyword, we screened for schizophrenia-related genes from the GeneCards, OMIM, PharmGKB, and Drug Bank databases. After removing duplicate genes with R software, we merged the results to obtain schizophrenia-related target genes. These were then matched with the gene targets of the LQDTD, and a Venn diagram was created to illustrate the overlaps.

### 2.5. Construction of the network

Using Cytoscape 3.7 software, we constructed an interaction regulatory network model with the candidate compounds, targets, and schizophrenia obtained from the LQDTD as nodes.

### 2.6. Protein–protein interaction analysis

The intersecting genes were imported into the STRING platform to construct the PPI network. The protein species was set to “Homo sapiens,” and the minimum interaction threshold was set to “highest confidence 0.9.” The network display option was configured to “hide disconnected nodes in the network,” while other parameters were left at their default settings, resulting in the in vivo response network of the LQDTD for the treatment of schizophrenia.

### 2.7. Gene enrichment analysis

The DAVID database was used to analyze the gene ontology (GO) enrichment of the target genes of the LQDTD. The Kyoto Encyclopedia of Genes and Genomes (KEGG) pathways enrichment analysis was carried out by R software, and enrichment analysis results were visually analyzed to clarify the main pharmacological mechanism of the treatment of schizophrenia.

### 2.8. Molecular docking

AutoDock 1.5.6 software was used to perform molecular docking of the 15 core genes identified. The affinities were all found to be less than −5 kJ/mol, indicating a strong binding energy between the drug components and the targets. A molecular docking mode diagram of the target protein and the corresponding compound was constructed, as illustrated in Figure 7.

**Figure 2.**
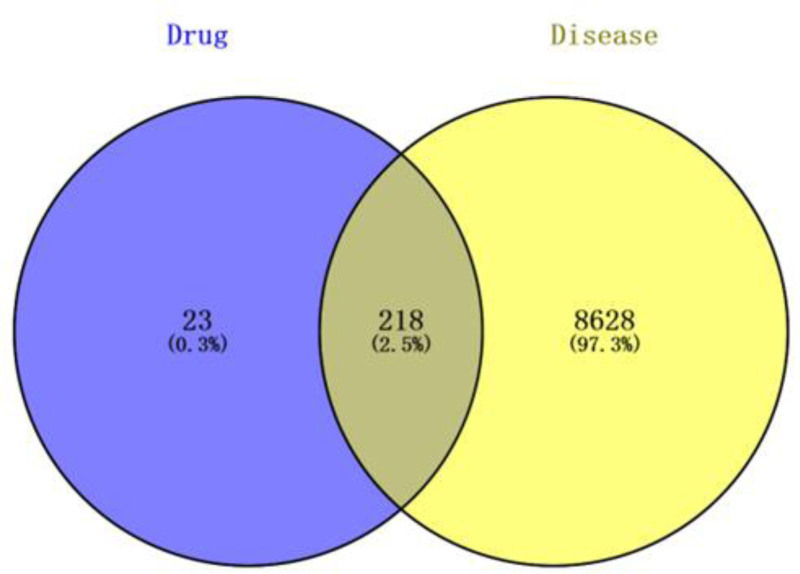
Shared targets of LQDTD and schizophrenia disease. LQDTD = Li Qi Dao Tan Decoction.

**Figure 3.**
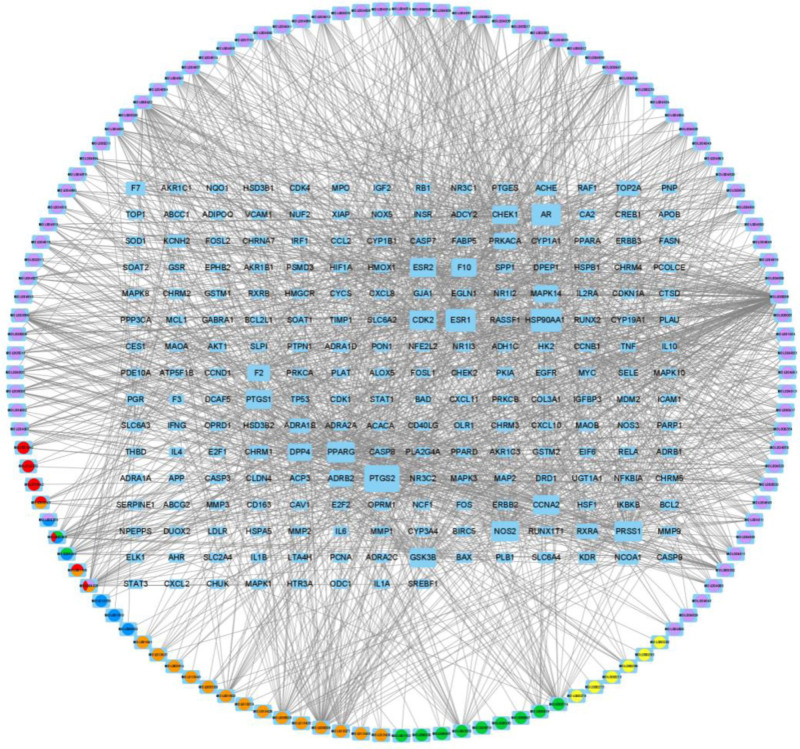
Network diagram of LQDTD-component – target – schizophrenia. LQDTD = Li Qi Dao Tan Decoction.

**Figure 4.**
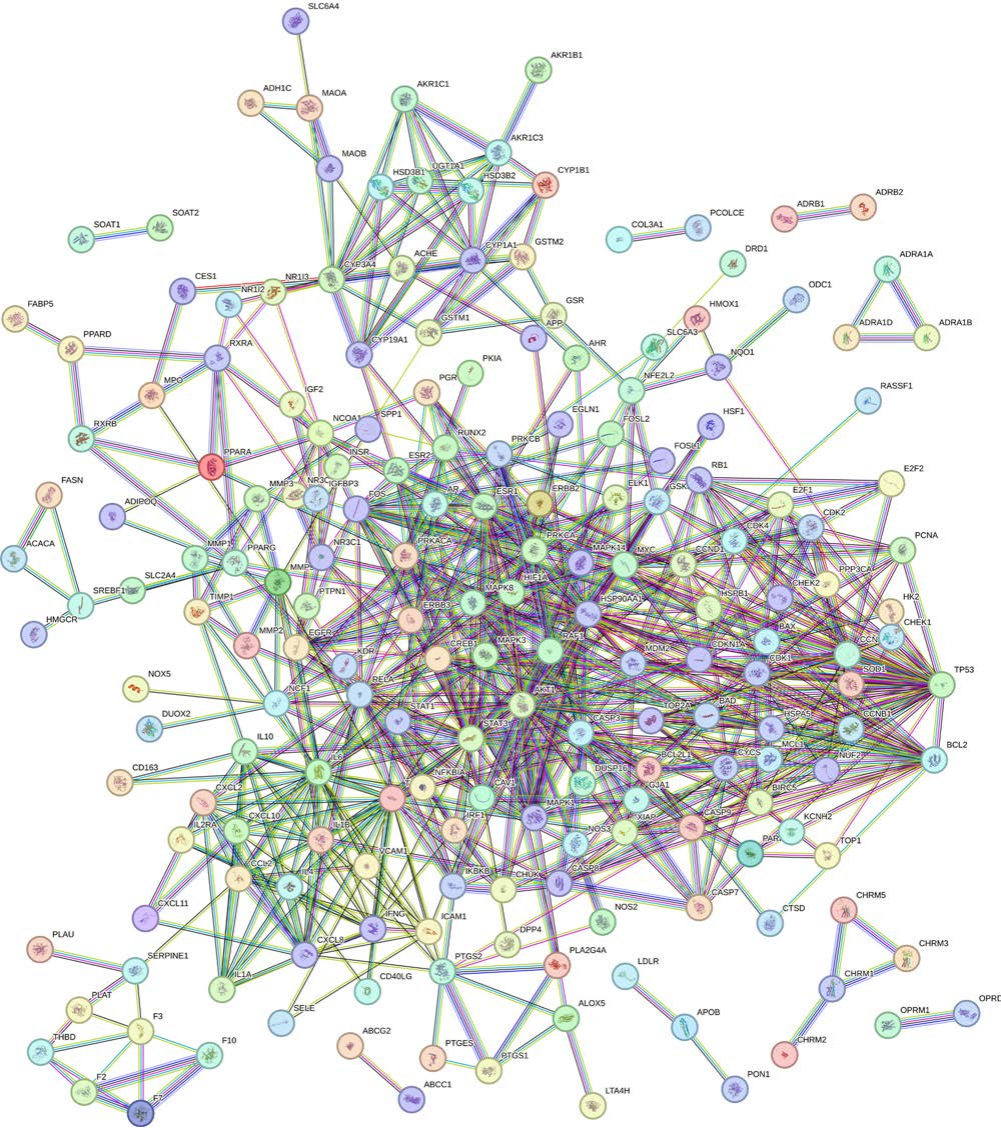
PPI network of common protein targets of LQDTD. LQDTD = Li Qi Dao Tan Decoction.

**Figure 5.**
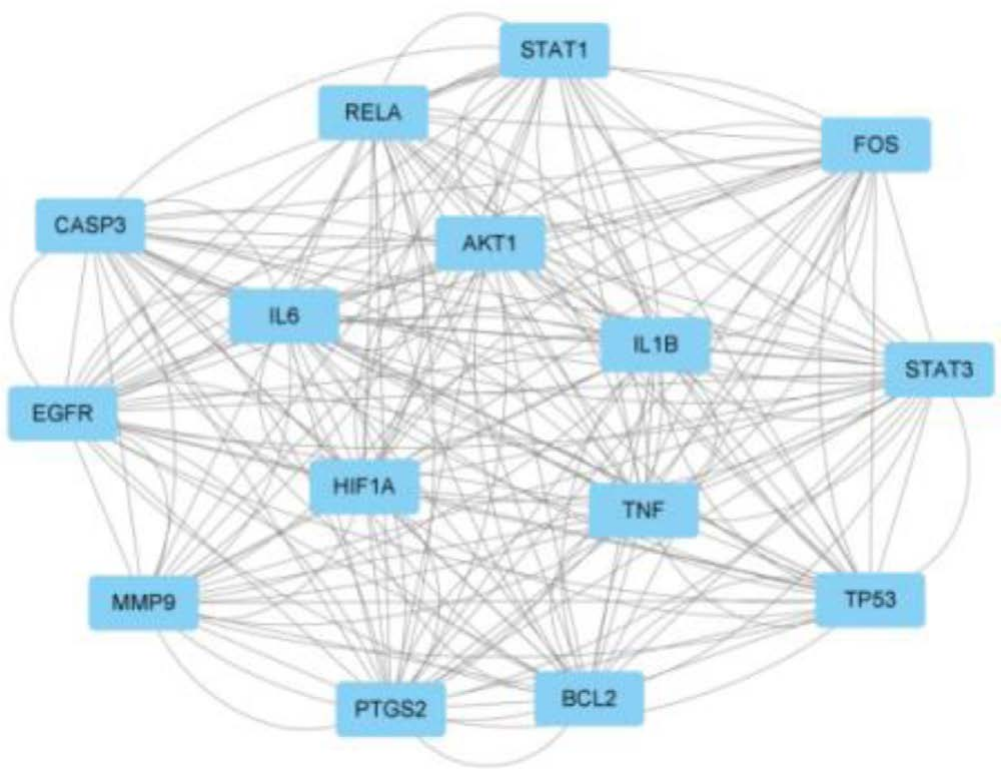
Key targets.

**Figure 6.**
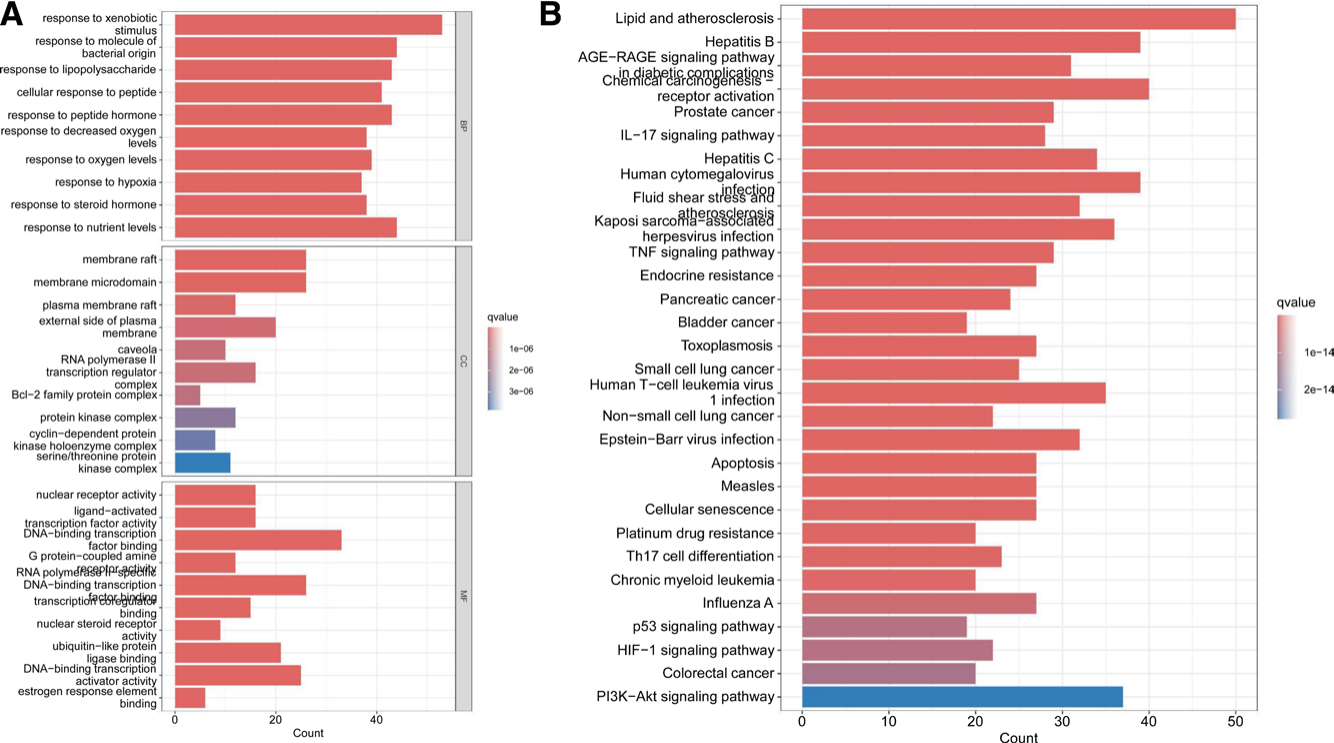
(A) GO enrichment analysis (top 10); (B) KEGG pathway enrichment analysis (top 30). GO = gene ontology, KEGG = Kyoto Encyclopedia of Genes and Genomes.

**Figure 7.**
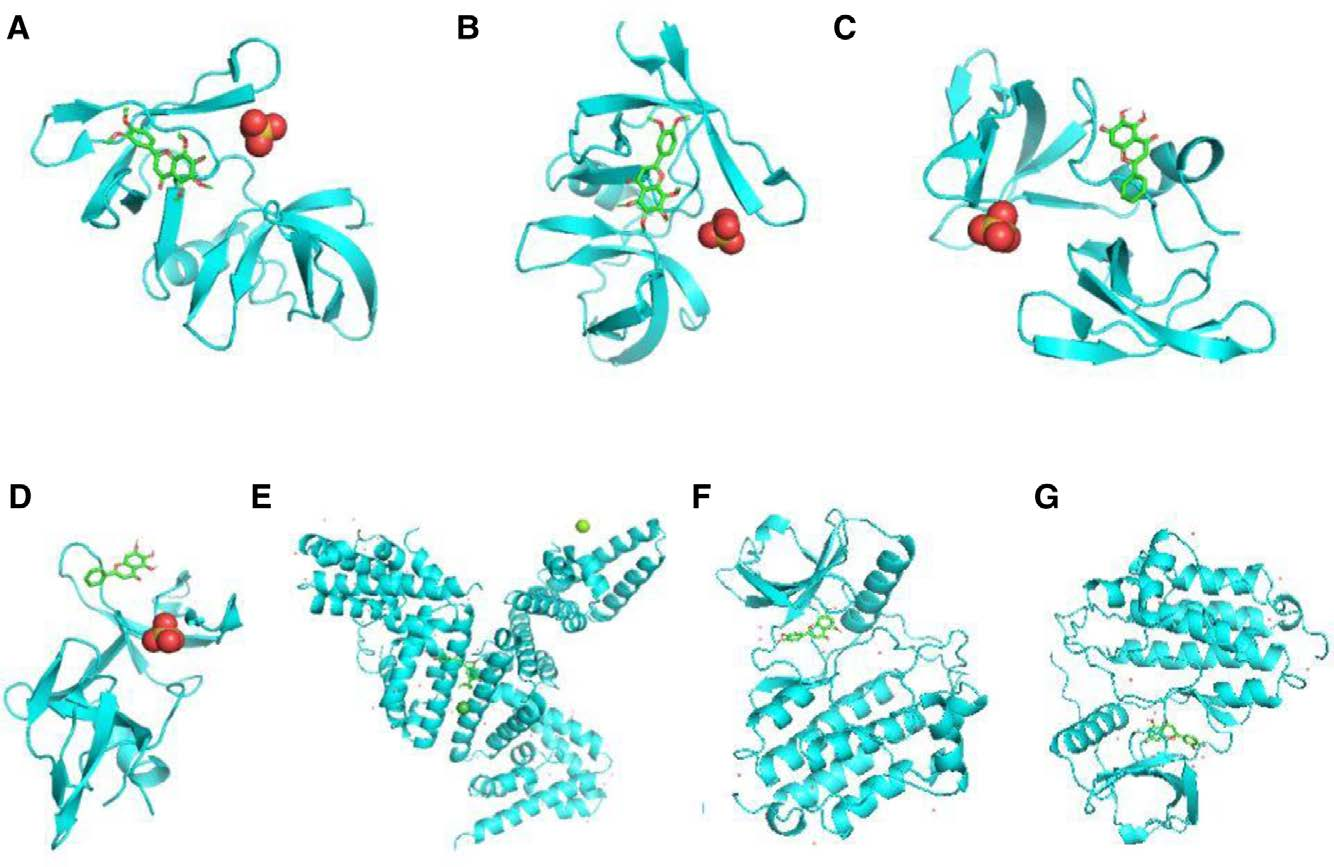
Molecular docking pattern diagram represents: (A) TP53 and nobiletin; (B) TP53 and quercetin; (C) TP53 and luteolin; (D) TP53 and baicalin; (E) STAT3 and licochalcone A; (F) EGFR and luteolin; (G) EGFR and quercetin. EGFR = epidermal growth factor receptor, STAT3 = signal transducer and activator of transcription 3.

## 3. Results

### 3.1. Screening of chemical constituents in 6 traditional Chinese medicines within LQDTD components

Utilizing the TCMSP database, 116 chemical components were identified in Pinelliae Tuber, 123 in Arisaematis Rhizoma, 34 in Poria, 280 in Liquorice Root, 65 in Fructus Auranti Immaturus, and 29 in Red Tangerine Peel. In total, 241 components met the criteria for (oral bioavailability ≥ 30%) and (drug-likeness ≥ 0.18) based on the ADME parameter screening standards.

### 3.2. Prediction of shared targets between LQDTD and schizophrenia

A total of 241 potential targets were identified through the paired analysis of the target prediction model from the TCMSP database. Additionally, 8846 targets related to schizophrenia were sourced from the GeneCards, OMIM, and PharmGKB databases. By matching and intersecting these datasets, 218 targets relevant to schizophrenia treatment were obtained. An intersection map illustrating the LQDTD – target – schizophrenia relationships was created using Cytoscape software, where yellow represents schizophrenia targets, blue signifies drug targets, and gray indicates the overlap between the 2. For further details, refer to Figure 2.

### 3.3. Construction of the LQDTD – target – schizophrenia network

Based on the target information from the intersection of the LQDTD, an interaction regulation network model was constructed using Cytoscape 3.7.0 software, as depicted in Figure 3. In this model, the orange circle represents Fructus Aurantii Immaturus, the green circle denotes Pinelliae Tuber, the yellow circle signifies Poria, the red circle indicates Red Tangerine Peel, the purple circle represents Liquorice Root, and the blue circle corresponds to Arisaematic Rhizoma. The square nodes represent protein receptors, with node size proportional to the number of associated active ingredients. For further details, refer to Figure 3.

### 3.4. Protein–protein interaction (PPI) network analysis

Based on the target information from the intersection of LQDTD, the PPI network was derived from the STRING database. The PPI network was constructed by importing the intersecting target proteins into the STRING platform, as illustrated in Figure 4. In this figure, the nodes represent the target proteins, with a higher number of connections indicating stronger evidence for correlation between them. The PPI analysis revealed 218 nodes representing predicted target proteins and 696 edges illustrating the relationships among these targets, resulting in an average node degree of 6.39.

The values for “Degree,” “Betweenness,” “Closeness,” “Eigenvector,” and “Local Average Connectivity-based method (LAC)” were all greater than the median, leading to the identification of 15 core targets after 4 rounds of screening. These core targets include PTGS2, AKT1, signal transducer and activator of transcription 3 (STAT3), TP53, interleukin-6, EGFR, interleukin-1β, B-cell CLL/lymphoma 2, caspase-3, tumor necrosis factor (TNF), fos proto-oncogene, STAT1, RELA, HIF1A, and matrix metalloprotein, as depicted in Figure 5.

### 3.5. LQDTD – target – schizophrenia gene ontology (GO) analysis

GO analysis was conducted using the DAVID database’s biomolecular function annotation system to evaluate the treatment of schizophrenia by LQDTD. This analysis encompasses 3 branches: biological process (BP), cellular component (CC), and molecular function (MF). Based on a significance threshold of *P* < .05, the top 10 items for BP, CC, and MF were selected for visualization in a histogram (Fig. 6).

The GO analysis revealed 2482 BP-related functional items, primarily including responses to xenobiotic stimuli, cellular responses to peptides, responses to molecules of bacterial origin, and responses to peptide hormones, as well as reactions to lipopolysaccharides, among others. There were 99 CC-related entries, mainly encompassing membrane rafts, membrane microdomains, plasma membrane rafts, the external side of the plasma membrane, and caveolae associated with RNA polymerase II. Additionally, 258 MF-related entries were identified, primarily including G protein-coupled amine receptor activity, DNA-binding transcription factor binding, nuclear receptor activity, ligand-activated transcription factor activity, and RNA polymerase II specificity.

### 3.6. KEGG pathway analysis of the LQDTD – target – schizophrenia

Through KEGG pathway analysis of 218 target genes associated with the treatment of schizophrenia by LQDTD, a total of 199 signaling pathways were identified. The results indicated that the potential genes were primarily enriched in pathways related to lipid and atherosclerosis, hepatitis B virus, AGE-RAGE signaling in diabetic complications, chemical carcinogenesis receptor activation, and the prostate cancer signaling pathway, among others. The top 30 pathways from this analysis are illustrated in a histogram (Fig. 6).

### 3.7. Molecular docking

The molecular docking analysis of the 15 core genes revealed that the affinities between STAT3, TP53, and EGFR and their corresponding active ingredients – luteolin, nobiletin, baicalin, quercetin, and licochalcone A – were all less than −5 kJ/mol (Table 1). This indicates a strong binding capacity between the drug components and the targets. The molecular docking pattern diagram illustrating the interactions between the target proteins and their corresponding compounds is presented in Figure 7.

**Table 1:** Molecular docking results.

Active ingredient	Target	Binding energy (kJ/mol)
Licochalcone A	STAT3	−29.301
Nobiletin	TP53	−27.208
Quercetin	TP53	−31.812
Baicalein	TP53	−31.394
Luteolin	TP53	−32.231
Luteolin	EGFR	−32.231
Quercetin	EGFR	−30.557

EGFR = epidermal growth factor receptor, STAT3 = signal transducer and activator of transcription 3.

## 4. Discussion

In TCM, schizophrenia is categorized under “madness,” while epilepsy is most commonly associated with the phlegm stagnation type. Additionally, the phlegm and fire internal disturbance type is more frequently observed in cases of madness.^[11]^ The positive symptoms of schizophrenia primarily manifest as hallucinations, delusions, agitation, hostility, and other similar states, aligning with the pathogenesis of madness in TCM. In contrast, the negative symptoms are characterized by apathy, impaired thinking, diminished will, lack of interest in social interactions, and attention deficits.^[12]^ Its pathogenesis is primarily attributed to qi stagnation and phlegm accumulation, leading to clouded consciousness.

TCM for the treatment of schizophrenia primarily utilizes remedies associated with the heart, lung, and spleen meridians.^[11]^ In this study, the formulation consisted primarily of 6 TCMs: Pinelliae Tuber, Arisaematis Rhizoma, Poria, Licorice Root, Fructus Aurantii Immaturus, and Red Tangerine Peel. These ingredients are clinically applicable for treating the internal obstruction of phlegm-dampness syndrome in schizophrenia.

Liquorice Root is used to tonify the spleen and replenish qi, clear heat, dispel phlegm, and harmonize various medicinal ingredients. It is effective in treating spleen deficiency and fatigue, heart deficiency and palpitations, as well as cough, wheezing, and acute limb pain. Additionally, licochalcone A, a compound uniquely found in Liquorice Root, exhibits notable analgesic, anti-inflammatory, and immune-regulatory effects.^[13–16]^ Poria is known to regulate neurotransmitters such as GABA, serotonin (5-HT), and dopamine (DA), thereby influencing the central nervous system.^[17]^ Pinellia Tuber is effective in drying damp phlegm, alleviating nausea and vomiting, and eliminating phlegm. The total alkaloids present in Pinellia Tuber exhibit neuroprotective effects and enhance spatial memory function.^[18]^ Research indicates that Frucutus Aurantii Immaturus is effective in breaking qi stagnation and dissipating accumulations, dissolving and dispersing phlegm. It also possesses potent expectorant and antianxiety properties.^[19]^ Red Tangerine Peel helps dissolve phlegm and relieve cough, while also regulating qi and supporting the spleen and stomach. Its components, such as hesperidin and neoeriocitrin, exhibit strong inhibitory effects on DNA oxidative damage.^[20]^

In this study, the potential active ingredient luteolin was identified, which not only enhances memory but also increases levels of brain-derived neurotrophic factor (BDNF) and acetylcholine.^[21]^ Nobiletin exhibits anti-inflammatory, antioxidant, and neuroprotective effects. It stimulates autophagy, inhibits acetylcholinesterase (AChE) activity, and reduces neuroinflammation and oxidative stress through the SIRT1/FoxO3a pathway. These properties provide strong evidence for the therapeutic potential of nobiletin in the treatment of Alzheimer’s disease.^[22]^ Baicalin inhibits acetylcholine (ACh) or ciclopirox (CPA)-induced endothelial/nitric oxide-dependent relaxation.^[23]^ Licochalcone A possesses neuroprotective effects by acting as a PTP1B inhibitor, enhancing cognitive function through the BDNF-TrkB pathway. It also inhibits microglial activation, thereby reducing neuroinflammation. These neurotransmitters and neurotrophic factors are associated with the onset and progression of schizophrenia.^[24]^

The pathogenesis of schizophrenia is believed to involve abnormal immune and inflammatory responses. Multiple studies have established a potential causal relationship between inflammation and brain structures, with dysregulation of the inflammatory response triggering cascades that impact neuronal development and, consequently, lead to downstream behavioral phenotypes.^[25]^ The PTGS2 gene encodes cyclooxygenase-2 (COX-2), a rate-limiting enzyme in prostaglandin synthesis that uniquely regulates inflammation. Additionally, the AKT1 gene polymorphism rs1130214 has been linked to antidepressant response in patients with depression.^[26]^ Elevated levels of interleukin-6 are associated with an increased risk of schizophrenia in offspring.^[27]^ The close relationship among these factors in the onset and progression of schizophrenia still requires experimental verification. Studies have indicated that the expression of B-cell CLL/lymphoma 2 and caspase-3 genes in patients with schizophrenia is significantly higher than in the control group, suggesting an intensification of the apoptotic process. This finding aligns with the theory of increased apoptosis in the pathophysiology of schizophrenia.^[28]^ TNF-alpha (TNF-α) plays a significant role not only in immune responses but also in the normal functioning of the central nervous system. Studies have shown that treatment with barrenwort may enhance spatial learning and memory in rats with schizophrenia through the TNF signaling pathway.^[29]^ The protein p53, encoded by the TP53 gene, is implicated in dopamine-induced apoptosis of neurons and astrocytes through its oxidative metabolites, such as glutamate or nitric oxide. It also plays a role in the development of oligodendrocytes and is considered a candidate gene for features related to myelin integrity in schizophrenia.^[30]^ STAT3 is involved in critical physiological and pathological processes, including cell proliferation, differentiation, survival, apoptosis, transformation, and cellular immunity, and is classified as a proto-oncogene.^[31]^ Additionally, mutations in the RELA gene may be linked to prepulse inhibition and startle response in schizophrenia.^[32,33]^

The PPI network was built based on the interactions of BDNF with immune (cytokines, STAT3), neurotrophic (NTRK2, NGFR, and NTF4), and cell-cell junction DEPs (differentially expressed proteins). Shared between bipolar disorder and major depressive disorder were the NRF2 pathway and signaling by the EGFR pathways.^[34]^ Other results indicate that TP53 was significantly associated with schizophrenia.^[35]^ Shen et al elevated levels of tumor necrosis factor (TNF) were observed in patients with schizophrenia, while the expression of tumor protein p53 (TP53) was significantly reduced.^[36]^ Previous meta-analyses have shown that patients with schizophrenia have a reduced risk of developing prostate cancer, suggesting that certain prostate cancer gene variants may provide a protective effect against schizophrenia. However, Mendelian randomization analysis does not appear to support a direct link between schizophrenia and prostate cancer.

GO enrichment analysis of the intersecting target genes revealed that BPes play a significant role in the treatment of schizophrenia with LQDTD, while MF and cellular composition accounted for a relatively smaller proportion. KEGG analysis indicated that the treatment of schizophrenia primarily operates through lipid and atherosclerosis pathways, hepatitis B virus-related pathways, AGE-RAGE signaling pathways, oncogenic chemical receptor activation, and prostate cancer signaling pathways.

Individuals with schizophrenia are at a higher risk of contracting the hepatitis B virus, even among those who have received routine vaccinations. They are approximately 3 times more likely to develop hepatitis B and C compared to healthy individuals. This association may be attributed to an increased prevalence of environmental risk factors, heightened susceptibility to infections, or a combination of both.^[37]^ It can be speculated that there are common susceptibility genes and molecular mechanisms shared between schizophrenia and conditions such as cancer and viral hepatitis, which aligns with the conclusions of previous studies.^[38,39]^ Recent studies identified the potential role of lipid peroxidation products as markers of oxidative stress and biomarkers of human diseases. Some authors have nicely described recent clinical data supporting the involvement of lipid peroxidation aldehydes in bipolar, schizophrenia, and other major depressive disorders.^[40]^ Schizophrenia has been linked to an elevated cardiovascular risk profile and premature onset of cardiovascular disease. Previous research indicates higher pericardial adipose tissue in individuals with schizophrenia compared to nonschizophrenic counterparts.^[41]^ As for whether it can have an impact on patients with schizophrenia by using the LQDTD, further experimental verification is still needed.

In summary, this study employed network pharmacology to construct an active ingredient-target protein-pathway network linking LQDTD and schizophrenia. It analyzed the interactions among the intersecting targets, identified core targets, and utilized molecular docking to verify the interactions between these core targets and their corresponding components, thereby predicting the mechanism of LQDTD in treating schizophrenia. The 5 active ingredients – luteolin, baicalin, quercetin, licochalcone A, and injectable quercetin – may act on multiple targets such as STAT3, TP53, EGFR, fos proto-oncogene, and matrix metalloprotein. They intervene in pathways related to lipid and atherosclerosis, hepatitis B virus, AGE-RAGE signaling, oncogenic chemical receptor activation, and prostate cancer signaling. Additionally, they respond to external stimuli, molecular responses to bacterial infections, and cellular responses to peptides, including reactions to peptide hormones, lipopolysaccharides, and various biological functions such as G protein-coupled receptor activity, DNA transcription factor binding, nuclear receptor activity, ligand-activated transcription factor activity, and RNA polymerase specificity.

Ancient herbal therapies are regaining popularity in disease management due to their natural origins, fewer side effects, and cost-effectiveness.^[42]^ Some studies have confirmed that some herbal medicines may be efficient for regulating the metabolic changes related to atypical antipsychotics due to their multipotential action, and more efforts should be made to make herbal drug treatments more effective.^[43]^ KangPiLao decoction (KPLD), which is composed of 6 Chinese herbal medicines, has been widely applied in clinical treatment, and some of which have confirmed anti-fatigue effects. KPLD significantly improved cognitive and emotional disorders in rats with CF by regulating the GABA/Glu pathway.^[44]^ Ayurveda has a well-developed area of psychiatry, with scientifically validated methods for treating mental and physical problems related to psychological imbalance. For various individuals’ mental problems, such as Alzheimer’s disease, Parkinson’s disease, depression, epilepsy, schizophrenia, anxiety, and others, herbal and Ayurvedic remedies are favored over synthetic medications.^[45]^ Apomorphine, luteolin, apigenin, caryophyllene, cannabinoids, baicalin, and reserpine are some of the phytochemicals that have shown antischizophrenic activity in human research.^[40]^ In our research, we also discovered the significant role of luteolin in pharmacology.

This study reflects the characteristics of multi-component, multi-target, and multi-pathway approaches, offering a novel perspective for treating schizophrenia with LQDTD. However, there are still limitations associated with network pharmacology. Firstly, the material basis research mostly focuses on the individual herbs in the prescription, while the research on the effective component group of the overall prescription is lacking; secondly, there is a lack of relevant pharmacological mechanism experimental research. Only a few animal experiments have been reported. Therefore, it is extremely necessary to study the mechanism and mode of action of this prescription in exerting clinical efficacy. Molecular docking and PPI network topological analysis (degree values, betweenness centrality) can be used to screen for high-confidence targets, reducing false positives. In subsequent studies, we can employ random grouping and strict inclusion/exclusion criteria to control baseline confounding variables. Some studies did not elaborate on unmeasured confounding factors (such as environment, genetic background), and further confirmation of the mechanism is needed through experimental verification. In addition to the existing literature, some findings of this study require validation through relevant experiments to provide a theoretical foundation for in-depth research on the key mechanisms of the LQDTD in the treatment of schizophrenia in the future.

Wen, Z. et al found that both cytokines and growth factors are capable of inducing the serine phosphorylation of STAT1 and STAT3.^[44]^ Thrombospondin 1 (THBS1) knockdown suppressed cell proliferation, migration, and invasion while enhancing cell apoptosis through the JAK2/STAT3 signaling pathway.^[45]^ Tetrahydrocurcumin (THC) induces the apoptosis of HCC cells through the TP53 signaling pathway, thereby inhibiting their proliferation and migration.^[46]^ In the future, we will also add cell experiments for verification.

In summary, this study investigated the potential key genes and molecular mechanisms of the LQDTD in the treatment of schizophrenia through network pharmacology, aiming to provide a new perspective for schizophrenia treatment and serve as a reference for further exploration of its underlying mechanisms. However, the relevant information included in the database is derived from various experimental conditions, and the interactions of TCM during compatibility may generate new components. Consequently, the components analyzed may not accurately reflect those that act on the human body. Further research on drug repositioning and TCM network toxicology still requires additional verification.

## 5. Conclusion

To the best of our knowledge, this is the first study to report using network pharmacology methods to systematically analyze LQDTD, identify its potential therapeutic targets, and provide a preliminary elucidation of its mechanism of action in treating schizophrenia. This approach is crucial for the comprehensive exploration of LQDTD’s therapeutic mechanisms. However, these findings were derived solely from network-prediction analysis and preliminary experimental validation; thus, further experimental verification of the results is required.

## Author contributions

**Data curation:** Chen Zhao.

**Formal analysis:** Gezi Shi.

**Methodology:** Shichang Yang.

**Visualization:** Jing Liu.

**Writing – original draft:** Shuo Wang.

**Writing – review & editing:** Fei Guo.
